# Supplementary material for: miR-7-5p and Importin-7 Regulate the p53 Dynamics and Stability in Malignant and Benign Thyroid Cells
Source: Int J Mol Sci. 2025 Jun 17;26(12):5813. doi: 10.3390/ijms26125813 (PMC12192917; doi:10.3390/ijms26125813)
Supplement: Supplementary file 1 [file ijms-26-05813-s001.zip › Table S1.docx]

**Table S1: Expression of TP53 and related molecules by RT-PCR in thyroid samples.**

|  | **Mean expression value ΔCq (SD)** | | | **NIFTP vs. FND** | **PTC vs. FND** |
| --- | --- | --- | --- | --- | --- |
|  | **FND (n=10)** | **NIFTP (n=11)** | **PTC (n=30)** | **Fold Change** | **Fold Change** |
| **TP53** | -0.98 (2.38) | -1.00 (1.31) | -0.60 (3.59) | 1.01 | **-1.30** |
| **MYC** | -1.91 (3.03) | -0.46 (0.03) | -1.19 (3.73) | **2.72-** | **-1.64** |
| **NFƙB** | 0.85 (2.67) | -0.13 (1.74) | 1.73 (4.65) | **1.97** | **-1.85** |

Fold change is calculated using the 2^-(ΔΔCq)^ formula. No statistical significance was detected.
