# Supplementary material for: miR-7-5p and Importin-7 Regulate the p53 Dynamics and Stability in Malignant and Benign Thyroid Cells
Source: Int J Mol Sci. 2025 Jun 17;26(12):5813. doi: 10.3390/ijms26125813 (PMC12192917; doi:10.3390/ijms26125813)
Supplement: Supplementary file 1 [file ijms-26-05813-s001.zip › Table S2.docx]

**Table S2: Expression of IPO7 and miR-7-5p in primary cultured PTC cells treated with IPO7 siRNA.**

| **Samples** | | **IPO7 ∆Cq** | **Fold change** | **MiR-7-5P ∆Cq** | **Fold change** |
| --- | --- | --- | --- | --- | --- |
| **1** | **IPO7 siRNA** | 4.23 | -108.38 | 9.25 | 8.28 |
|  | **Control** | -2.53 |  | 12.3 |  |
| **2** | **IPO7 siRNA** | 3.45 | -17.87 | 0.73 | 37.27 |
|  | **Control** | -0.71 |  | 5.95 |  |
| **3** | **IPO7 siRNA** | 2.75 | -23.26 | 5.38 | 12.55 |
|  | **Control** | -1.79 |  | 9.03 |  |
| **4** | **IPO7 siRNA** | 5.17 | -171.25 | -1.43 | 910.17 |
|  | **Control** | -2.25 |  | 8.4 |  |
| **5** | **IPO7 siRNA** | 4.25 | -126.23 | 4.35 | 4.76 |
|  | **Control** | -2.73 |  | 6.6 |  |
|  |  |  | P< 0.001 |  | P<0.001 |
|  |  |  |  |  |  |
|  |  |  |  |  |  |
