# Supplementary material for: miR-7-5p and Importin-7 Regulate the p53 Dynamics and Stability in Malignant and Benign Thyroid Cells
Source: Int J Mol Sci. 2025 Jun 17;26(12):5813. doi: 10.3390/ijms26125813 (PMC12192917; doi:10.3390/ijms26125813)
Supplement: Supplementary file 1 [file ijms-26-05813-s001.zip › Table S3.docx]

| **Samples** | | **IPO7 ∆Cq** | **Fold change** |
| --- | --- | --- | --- |
| Sample 1 | Treated | -0.41 | 12.55 |
|  | Control | 3.24 |  |
| Sample 2 | Treated | 1.3 | 4.00 |
|  | Control | 3.3 |  |
| Sample 3 | Treated | -0.64 | 2.68 |
|  | Control | 0.78 |  |
|  |  |  | P<0.05 |

**Table S3: miR-7-5p loss of function effect on IPO7 expression in NTHY-ORI cells.**
