# Supplementary figures and images for: miR-7-5p and Importin-7 Regulate the p53 Dynamics and Stability in Malignant and Benign Thyroid Cells
Source: Int J Mol Sci. 2025 Jun 17;26(12):5813. doi: 10.3390/ijms26125813 (PMC12192917; doi:10.3390/ijms26125813)

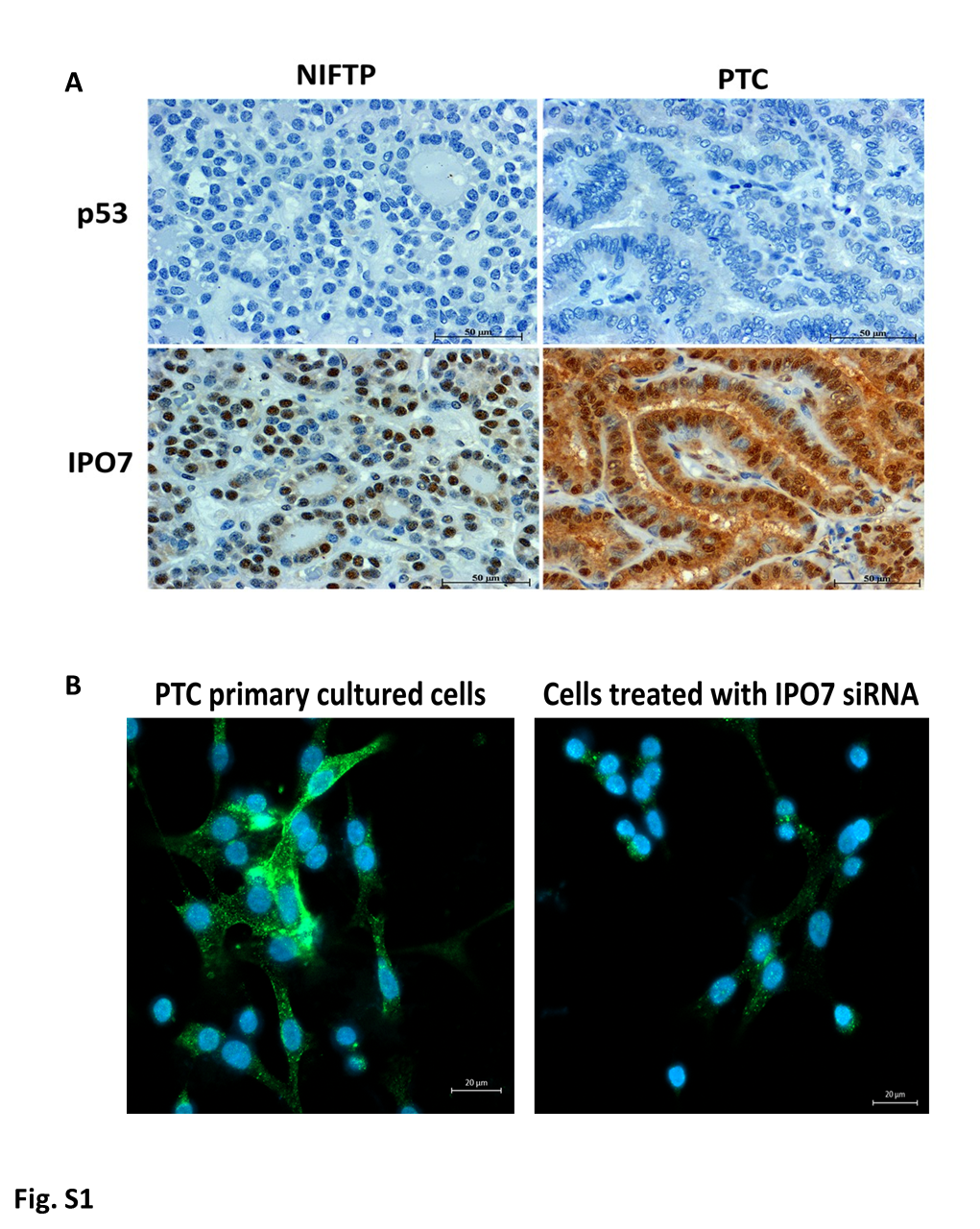

Supplement: Supplementary file 1 [file ijms-26-05813-s001.zip › Fig. S1.tif]

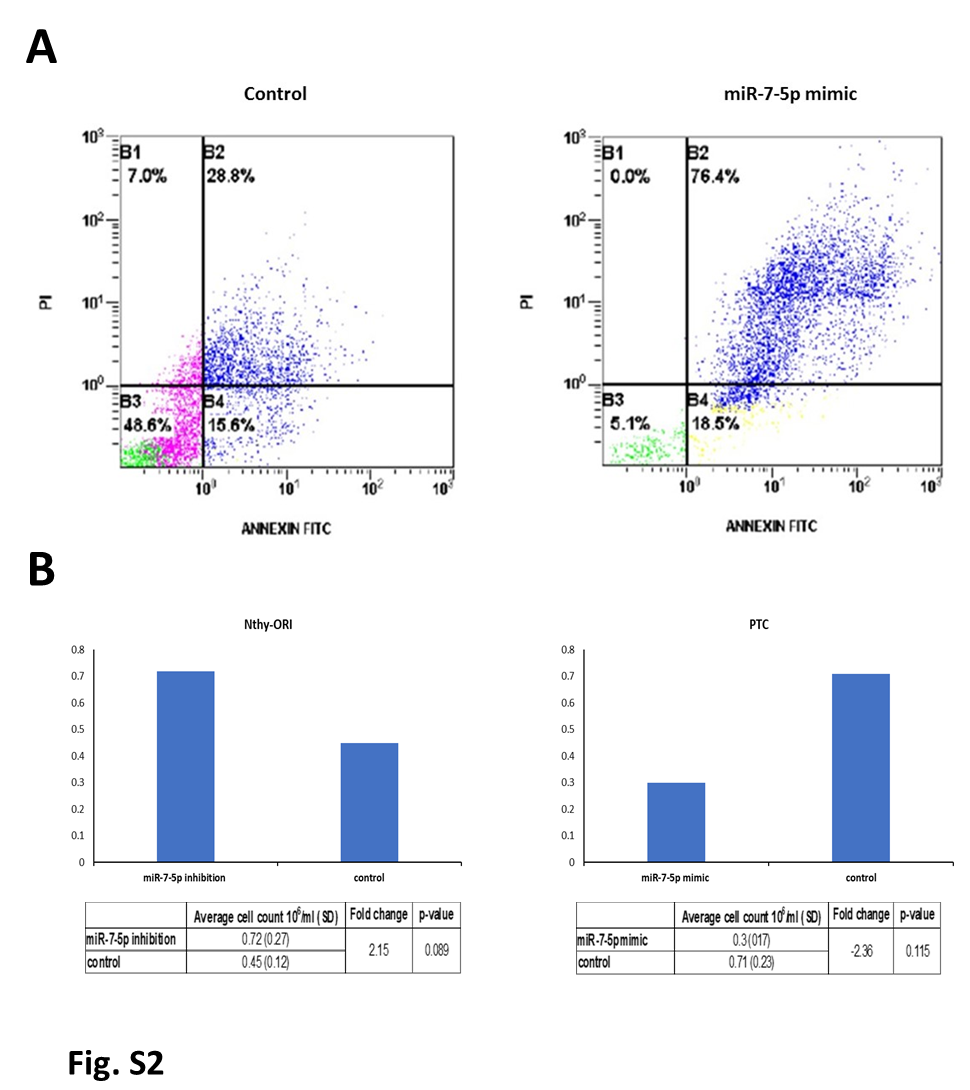

Supplement: Supplementary file 1 [file ijms-26-05813-s001.zip › Fig. S2.tif]

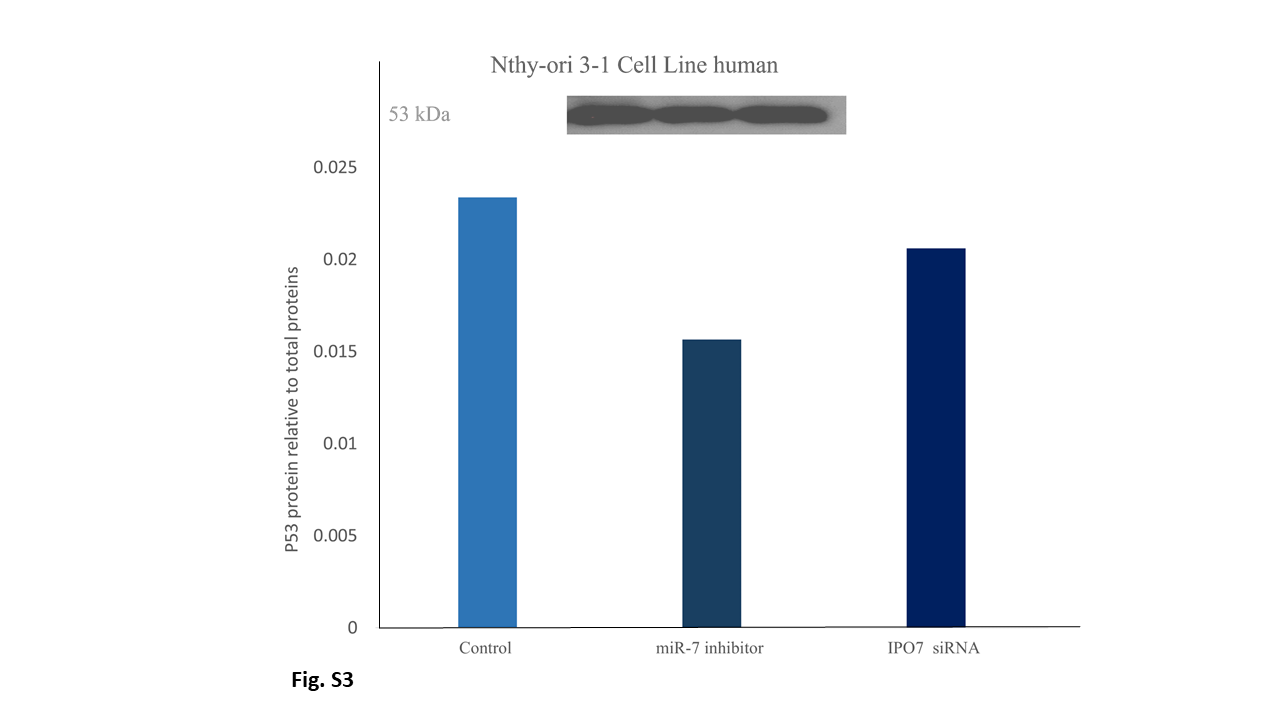

Supplement: Supplementary file 1 [file ijms-26-05813-s001.zip › Fig. S3.tif]
